# In Vivo Recovery of Bacteriophages and Their Effects on *Clostridium perfringens*-Infected Broiler Chickens

**DOI:** 10.3390/vetsci9030119

**Published:** 2022-03-07

**Authors:** Hyun-Gwan Lee, Yoo-Bhin Kim, Sang-Hyeok Lee, Jun-Ok Moon, Jong-Pyo Chae, Yu-Jin Kim, Kyung-Woo Lee

**Affiliations:** 1 Department of Animal Science and Technology, Konkuk University, Seoul 05029, Korea; leehyun3177@naver.com (H.-G.L.); ybin51@naver.com (Y.-B.K.); the_shinism@naver.com (S.-H.L.); 2 CJ Cheiljedang Co., Ltd., Suwon 16495, Korea; junok.moon@cj.net (J.-O.M.); jp.chae@cj.net (J.-P.C.); yj.kim20@cj.net (Y.-J.K.)

**Keywords:** *Clostridium perfringens*, bacteriophage, broiler chicken, gut health

## Abstract

The objectives of this study were to recover bacteriophages (BPs) from the intestinal digesta of BP-fed broilers and to evaluate the antibacterial effects of encapsulated or powdered BPs in broiler chickens challenged with *Clostridium perfringens*. Day-old broiler chicks (*n* = 320/experiment) were randomly assigned to 32 pens (*n* = 10 broilers/pen) and allocated to one of four dietary groups: (1) unchallenged group (NEG); (2) *C. perfringens*-challenged group (POS); (3) POS group fed a diet supplemented with powdered BPs; and (4) POS group fed a diet supplemented with encapsulated BPs. On days 21, 22, and 23 post-hatch, all chickens except NEG were orally inoculated twice a day with 2 mL *C. perfringens* (1.0 × 10^8^ cfu/mL). Varying BP levels were detected in gut digesta at all ages and were numerically or significantly higher in the encapsulated BP group than in the powdered BP group. Dietary powder or encapsulated BPs reversed the *C. perfringens*-mediated increase in crypt depth. In addition, villus height to crypt depth ratio was elevated in the NEG and BP-treated/challenged groups compared with that in the POS group. *C. perfringens* counts in the cecum were significantly lower in the BP-fed chickens than in the POS group. The encapsulated BP-supplemented diet-fed chickens had the highest serum IgA levels. Collectively, our results suggest that dietary BP remains viable in intestinal digesta upon ingestion and can inhibit cecal *C. perfringens* counts.

## 1. Introduction

Necrotic enteritis (NE), an economically important enteric disease in the broiler industry, is caused by gram-positive anaerobic *Clostridium perfringens* type A/G that impacts the global industry by compromising the performance, health, and welfare of chickens [1,2]. *C. perfringens* toxinotypes are classified from A to G depending on their production capability for α-toxin, β-toxin, ε-toxin, ι-toxin, enterotoxin, and NE beta-like (NetB) toxin [2]. *C. perfringens* typically contains approximately 10^4^ colony-forming units (cfu) per gram of digesta in the intestine of healthy birds [3]. *C. perfringens*/*Eimeria* dual-infection induces significant changes in the gut structure and microbiome, allowing pathogenic *C. perfringens* to reach a critical concentration leading to NE [4,5]. Due to the ban on or voluntary phase-out of antibiotic growth promoters in Europe and many other countries, NE has re-emerged in the poultry industry, causing an annual economic loss of 2–6 billion US dollars globally [6,7].

Consequently, alternative veterinary and nutritional methods, such as the use of bacteriophages (BPs), vaccines, or probiotics, have been proposed as potential strategies for preventing NE [8,9,10]. Among these alternatives, BP is an obligate parasite of bacteria that uses bacterial cells to replicate and has long been used in the fields of human and veterinary medicine, agriculture, aquaculture, and the food industry, with no safety concerns [11]. BP-expressed endolysins can destroy the peptidoglycan layer of pathogenic bacteria as novel antimicrobial agents [12], and BPs exhibit antibacterial effects against *C. perfringens* in broiler chickens [8,9]. BPs show antibacterial specificity against various bacteria, including colibacillosis, *Salmonella* gallinarum, *Campylobacter* and *C. perfringens*, in chickens [8,13,14,15].

However, high temperature/humidity in growing facilities and acidic environments in animal guts, which are often encountered during poultry production globally, are critical factors that negatively affect the stability, survival, and activity of BPs [16,17]. In addition, BPs have limited stability in solution, showing a significant drop in their antibacterial activity [18,19]. These findings suggest that dietary BPs may undergo degradation after contact with water-rich digesta upon ingestion.

Encapsulation or coating methods have been used to preserve BP stability [19,20]. Encapsulation technology may also help ensure the safety of dietary BPs [19], keeping them viable in gastric and intestinal digesta. To date, no studies have attempted to isolate BPs from the gut digesta in broiler chickens after ingestion of dietary BPs in an encapsulated or powder form. Thus, the objectives of this study were to evaluate the antibacterial effects of BPs, either in encapsulated or powder form, against *C. perfringens* and to isolate BPs from various segments of the gastrointestinal tract to assess their stability. To meet these objectives, broiler chickens were artificially inoculated with *C. perfringens* to evaluate the antibacterial effects of BP.

## 2. Materials and Methods

### 2.1. Ethical Approval

The experimental procedure was approved by the Institutional Animal Care and Use Committee of Konkuk University (KU20180-1).

### 2.2. Animals, Diets, and Experimental Design

A total of 320 1-day-old unsexed broiler chicks (Ross 308) were obtained from a local hatchery. Upon arrival, they were individually weighed and randomly placed into 32 floor pens with fresh rice husk as a bedding material. Broilers were assigned to one of four treatment groups with 8 replicate pens per treatment and 10 birds per pen. The four groups consisted of the unchallenged group (NEG), the challenged group (POS), and the POS groups fed diets supplemented with encapsulated or powdered BP.

A corn and soybean meal-based diet was used as the control diet (Table 1), and the experimental diets were formulated by mixing the control diet with either powder (10^6^ pfu/g of diet) or encapsulated (10^6^ pfu/g of diet) BPs. BPs were originally isolated from chicken feces [8] and encapsulated using hot-melt fluidized bed coating process. Encapsulated BP was found to be stable in harsh conditions, such as high humidity and temperature [19]. The recommended inclusion level of dietary BP at 1.0 × 10^6^ pfu/g of diet has been previously published [8]. Freshly prepared experimental diets were provided every 3 days, and the leftovers were weighed and discarded. This practice was adopted to accurately deliver viable BPs to broiler chickens. Experimental diets were sampled to quantify the intended BP counts and stored at –20 °C until use. BP was not detected in the non-supplemented control diets. Encapsulated and powdered BP diets were analyzed to have a mean recovery rate of 123% (1.23 × 10^6^ pfu/g) and 119% (1.19 × 10^6^ pfu/g), respectively.

The temperature of the facility was initially set at 32 °C during the first week, then gradually decreased to 23 °C at 21 days post-hatch, which remained constant thereafter. Feed and water were provided ad libitum throughout the 28-d-feeding trial, and light was provided for 23 h/day. Body weight and feed intake per pen were measured at the beginning and at 7, 14, 21, and 28 days of the experiment, and used to calculate the feed conversion ratio. Feed intake was corrected for mortality.

### 2.3. C. perfringens Challenge

**Figure 1:**
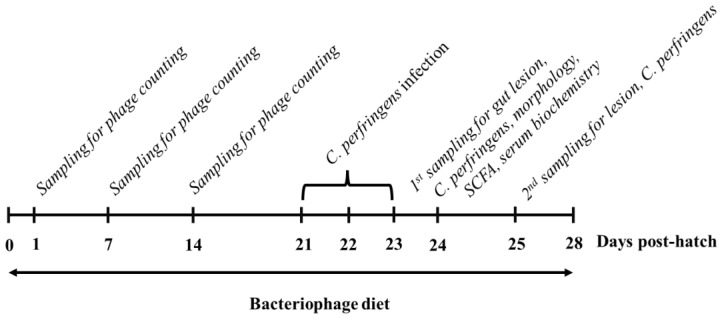
Experimental scheme. Broiler chicks were fed a control or bacteriophage (BP)-added diet for 28 d. On d 21, 22, and 23, half of the control group and BP-added diet-fed groups were orally inoculated with *Clostridium perfringens*. On d 1, 7, and 14, one bird per group (*n* = 8/group) was randomly sampled for BP isolation in gut digesta. On d 1 post *C. perfringens* challenge, one bird per group (*n* = 8/group) was sampled for gut lesion, *C. perfringens* counts, ileal morphology, cecal short-chain fatty acid (SCFA) contents, and serum biochemistry. On d 2 post *C. perfringens* challenge, one bird per group (*n* = 8/group) was sampled for gut lesion and *C. perfringens* counts.

On days 21, 22, and 23, broiler chickens were orally inoculated twice a day with 2 mL of either saline or *C. perfringens* CJ17 strain (1.0 × 10^8^ cfu/mL). The *C. perfringens* CJ17 strain harbored the NE B-like (NetB) gene [8] and was anaerobically incubated in fluid thioglycolate (Sigma-Aldrich, St. Louis, MO, USA) broth at 37 °C before oral gavage. The scheme of the experimental schedule is shown in Figure 1.

### 2.4. Sample Collection

On days 1, 7, and 14, one bird per replicate was randomly euthanized with an overdose of carbon dioxide and sampled to quantify or detect the presence of BP in various segments of the gastrointestinal tract. Immediately after euthanasia, the whole gastrointestinal tract from crop to ceca was excised and processed to collect the digesta from the crop, gizzard, jejunum, and ceca on the day of sampling.

On days 1 and 2 post *C. perfringens* challenge (24- and 25-days post-hatch), one bird per pen close to average body weight was selected and euthanized with an overdose of carbon dioxide. On day 1 post *C. perfringens* challenge, following euthanasia, blood was sampled in a clot activator tube (BD Vacutainer CAT Plus Blood Collection Tubes; Becton Dickinson, Plymouth, UK) by cardiac puncture. Serum samples were obtained by gentle centrifugation (200× *g*) for 15 min and stored at –20 °C before analysis. Immediately after blood sampling, the small intestine and a pair of ceca were aseptically sampled and processed for gut lesions, short-chain fatty acids, and *C. perfringens* counts on the same day of sampling. One day 2 post *C. perfringens* challenge, all post-mortem procedures were identical but only sampled for the small intestine and a pair of ceca to assay intestinal lesions and *C. perfringens* counts.

### 2.5. Bacteriophage Assay in the Feed and Gut Digesta

BP levels were measured in the feed and gut digesta sampled from the crop to the cecum. In brief, feed (ca. 100 g) and digesta (ca. 1 g) were homogenized in 10-fold volumes of the saline-magnesium (SM) buffer (G-Bioscience, St. Louis, MO, USA), followed by centrifugation at 10,000× *g* for 10 min, and the supernatants were filtered using a 0.25 µm filter. The filtrates were then serially diluted 10-fold with a sterilized SM buffer. The 10-fold diluted filtrates were quantified for BP titer using an agar overlay assay, as described [8]. Briefly, 100 µL of 10-fold diluted filtrates were added to 5 mL of 0.7% agar (*w*/*v*), and the mixtures were poured into a brain-heart infusion agar (Difco Laboratories, Detroit, MI, USA) plate with 0.2% sheep blood, allowed to harden, and incubated at 37 °C for 24 h. The viral titer of BP was expressed as plaque-forming units per gram of feed or digesta. On day 1, the jejunal and cecal digesta were pooled for BP quantification due to the insufficient gut volume. The detection limit of the assay is estimated to be 1.0 × 10^2^ pfu/g of sample.

### 2.6. C. perfringens Counts in the Cecal Digesta

Approximately 1 g of cecal digesta obtained on days 1 and 2 after *C. perfringens* challenge was mixed with 9 mL of sterile phosphate-buffered saline (PBS) and serially diluted 10-fold from 10^−2^ to 10^−4^. The 10-fold dilutions were then spiral-plated on tryptose-sulfite-cycloserine agar (TSC agar; Oxoid Ltd., Hampshire, UK) and incubated in an anaerobic cabinet at 37 °C for 24 h. The numbers of characteristic black colonies were counted and expressed as log_10_ cfu per gram of digesta.

### 2.7. Intestinal Lesion Score

On days 1 and 2 post *C. perfringens* challenge, one bird per pen was euthanized with an overdose of carbon dioxide. Immediately after euthanasia, approximately 30-cm long segments of the small intestine (15-cm before and after Meckel’s diverticulum) were excised and examined for the presence of NE lesions, if present, on a scale from 0 to 4, as previously described [21]. Three independent observers performed the examination and scoring in a blinded manner.

### 2.8. Intestinal Morphology

On day 1 post *C. perfringens* challenge, one bird per pen was euthanized with an overdose of carbon dioxide for the measurement of intestinal morphometry. A 1-cm long ileal mid-segment was fixed in 10% neutral-buffered-formalin for a minimum of 48 h. Samples were sectioned at 4.0 µm and mounted on slides for standard hematoxylin-eosin staining. Villus height and crypt depth were individually assessed in 8 well-oriented intact villi. Villus height was measured from the villus tip to the bottom, and crypt depth was measured from the bottom of the villus to the mucosa. Then, the ratio of villus height to crypt depth was calculated.

### 2.9. SCFA Analysis

On day 1 post *C. perfringens* challenge, approximately 1 g of cecal digesta was homogenized with 4 mL of ice-cold sterile PBS added with 0.05 mL of saturated HgCl2, 1 mL of 25% H3PO4, and 0.2 mL of 2% pivalic acid, centrifuged at 10,000× *g* at 4 °C for 20 min. One milliliter of supernatant was used to measure the concentrations of SCFA by gas chromatography (6890 Series GC System; HP, Palo Alto, CA, USA), as described previously [22].

### 2.10. Serum Parameters

Serum samples that had been obtained from day 1 post *C. perfringens* challenge were thawed on ice before biochemical analysis. Serum total antioxidant capacity (TAC) was analyzed using the QuantiChrom antioxidant assay kit (BioAssay System, Hayward, CA, USA) and expressed as Trolox equivalents. Nitric oxide (NO) levels were determined using the Griess reagent (Sigma, St. Louis, MO, USA), as described [23]. Serum immunoglobulin A (IgA) levels were determined using chicken IgA enzyme-linked immunosorbent assay (ELISA) (Bethyl Laboratories Inc., Montgomery, TX, USA). Serum corticosterone concentrations were assayed using a commercial Enzo Life Sciences Corticosterone ELISA kit (ADI-901-097; Enzo Life Sciences, New York, NY, USA). All analyses were performed according to the manufacturer’s instructions. In addition, serum biochemical parameters were analyzed for glutamic pyruvic transaminase (GPT), glutamic oxaloacetic transaminase (GOT), high-density lipoprotein (HDL) cholesterol, total cholesterol, and triglyceride levels using an automatic blood chemical analyzer (Film DRI CHEM 7000i, Fuji film, Tokyo, Japan).

### 2.11. Statistical Analysis

Each pen was considered an experimental unit. Data were analyzed by one-way analysis of variance using PROC GLM (version 9.4; SAS Institute Inc., Cary, NC, USA). Duncan’s multiple range test was used to determine the means and differences among treatments. The significance level was set at *p* < 0.05.

## 3. Results

### 3.1. BPs in Gut Digesta

**Figure 2:**
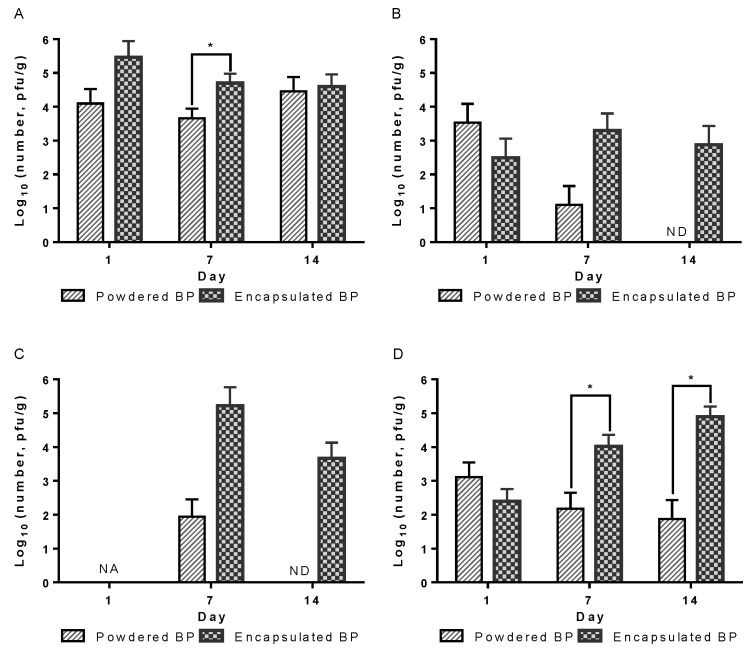
Viable BP levels in various segments of gastrointestinal tract. (**A**) Crop, (**B**) gizzard, (**C**) jejunum, and (**D**) cecum. Day-old broiler chickens were fed diets with or without encapsulated and powder BPs and sacrificed on different days for sampling gut digesta. Viable BPs (*Y*-axis) in figures are presented in log_10_ (pfu/g of digesta). Error bars represent the standard deviation of the mean. BPs were not detected in the no-BP-added diet-fed chickens (data not shown). Asterisks denote the significant differences in BP levels between the encapsulated and powder BP groups at the 0.05 level. NA = not assayed, ND = not detected.

As expected, no BPs were detected in the gut digesta obtained from the NEG and POS groups. However, varying BP levels in the BP-supplemented groups were detected in the gut digesta of different organs (Figure 2). BP levels ranged from 4.5 × 10^3^ to 2.9 × 10^5^ pfu/g in the crop, from none to 3.4 × 10^3^ pfu/g in the gizzard, from none to 1.7 × 10^5^ pfu/g in the jejunum, and from 7.5 × 10 to 8.0 × 10^4^ pfu/g in the cecum. BP levels in digesta at all ages were numerically or significantly higher in the encapsulated vs. powder BP groups, especially in the gizzard, jejunum, and cecum.

### 3.2. Growth Performance

It is clear from this study that both dietary BP, either encapsulated or powder form, and *C. perfringens* challenge had no effects (*p* > 0.05) on the production performance of broiler chicken (Table 2). Live body weight at 28 days ranged from 1801 to 1879 g per bird. Body weight gain following *C. perfringens* challenge at 21 to 28 days ranged from 731 to 783 g per bird (*p* > 0.05). Feed intake before and after *C. perfringens* challenge was not altered by dietary encapsulated or powdered BP groups at all ages. Finally, the feed conversion ratio was not affected (*p* > 0.05) by dietary BP or *C. perfringens* challenge and remained low at 0.93 to 0.98, 1.09 to 1.11, 1.21 to 1.23, and 1.31 to 1.39 at 1, 2, 3, and 4 weeks of age, respectively.

### 3.3. C. perfringens Counts in the Cecal Digesta

On day 24 post-hatch (i.e., day 1 post *C. perfringens* challenge), cecal *C. perfringens* counts ranged from 4.75 to 5.52 cfu per g of digesta and were significantly elevated (*p* = 0.008) in the POS vs. NEG groups (Table 3). In addition, both encapsulated and powdered BP equally lowered (*p* = 0.008) cecal *C. perfringens* counts compared with the POS group.

On day 25 post-hatch (i.e., day 2 post *C. perfringens* challenge), *C. perfringens* challenge significantly increased (*p* = 0.047) the cecal counts of *C. perfringens* and dietary BP reversed (*p* = 0.047) the challenge-induced increase in cecal *C. perfringens* counts. No difference between encapsulated and powdered BP groups on cecal *C. perfringens* counts was noted. No NE-specific gut lesions were observed in any of the treatment groups sampled on days 1 and 2 following *C. perfringens* challenge (data not shown).

### 3.4. Intestinal Morphology

Ileal morphology was examined in broilers at 24 days post-hatch (i.e., day 1 following *C. perfringens* challenge). *C. perfringens* challenge or dietary BP did not affect ileal villus height (*p* > 0.05) ileal villus height (Table 4). In contrast, *C. perfringens* increased ileal crypt depth (*p* = 0.004) compared to that in the NEG group. Dietary encapsulated and powdered BP decreased (*p* = 0.004) the *C. perfringens*-induced increase in ileal crypt depth (Table 4). Consequently, ileal villus height to crypt depth ratio decreased (*p* = 0.003) in the POS vs. NEG groups. Dietary BP restored (*p* = 0.003) the *C. perfringens*-induced decrease in ileal villus height to crypt depth ratio.

### 3.5. Concentration of SCFA in the Cecal Digesta

No effect of *C. perfringens* challenge or dietary BP on the concentrations of SCFA in cecal digesta was noted (*p* > 0.05; Table 5). The POS group had the lowest (*p* > 0.05) total SCFA compared with the NEG group and the BP-added diet-fed groups (i.e., encapsulated and powdered BP). However, *C. perfringens* challenge or dietary BP did not affect the relative percentages of SCFAs.

### 3.6. Serum Parameters

Table 6 summarizes the levels of various serum markers for antioxidant capacity (TAC), immunity (NO, IgA), stress (corticosterone), and metabolism (lipid metabolism or liver function). It was noted that *C. perfringens* challenge (POS group) lowered the concentrations of IgA in serum samples by 14.9% (on an average) compared with the NEG group. Conversely, dietary encapsulated and powdered BPs increased the serum IgA concentrations by 43.5% (*p* = 0.046) and 15.0% (*p* > 0.05), respectively, compared with the POS group. Except for IgA, none of the treatments affected the serum parameters, including TAC and NO, corticosterone, total cholesterol, HDL cholesterol, triglyceride, GPT, and GOT levels.

## 4. Discussion

BP has been a useful alternative to in-feed antibiotics in the poultry industry due to the evolution of multidrug-resistant bacteria, and it will be a major tool in the post-antibiotic era [12]. The potential advantage of BP in poultry industry is expected as BP can be used as a dietary supplement for healthy human individuals with mild to moderate gastrointestinal dysbiosis and is known to exhibit no or milder influence on commensal bacteria but to target multidrug-resistant pathogens [24,25]. The goals of this study were to demonstrate the ability of dietary BP to inhibit *C. perfringens* growth in chicken intestines and to examine their stability in various segments of the gastrointestinal tract when BP was delivered in either powder or encapsulated form.

Most BP species are acid-sensitive and cannot tolerate acidic conditions [17,26]. The lack of stability upon exposure to acidic conditions (e.g., in the proventriculus/gizzard) and the relatively short residence times in the intestinal tract may limit the efficacy of orally delivered BPs [17]. To overcome these obstacles, encapsulation is proposed to provide protection from gastric acidity, releasing high doses of diet-origin BP distally [27,28]. In this study, we confirmed that encapsulated BP effectively delivered viable BP distally, suggesting that the encapsulation or coating of BPs may further increase their stability and viability during storage and upon ingestion. The practical advantages of BP encapsulation can be realized in commercial production settings, where high humidity and temperature are commonly maintained.

*C. perfringens* is the major etiologic agent of NE in broiler chickens [9]. NE-afflicted chickens have damaged intestinal mucosa, leading to decreased growth rates and poorer feed efficiency [8,29,30]. It is well documented that *C. perfringens* per se does not induce NE, although it did increase the colonization of *C. perfringens* in the intestinal tract [31,32,33], which explains the lack of *C. perfringens* challenge on the production performance of chickens in this study. In line with our findings, *C. perfringens* had no effect on the growth performance of broiler chickens was found [34,35]. In an earlier study, we reported that *C. perfringens*/*Eimeria* spp. dual challenge induced clinical NE in chickens, leading to increased mortality and decreased growth performance [8]. Dietary powdered BP at 10^7^ pfu/kg significantly improved the health and performance of NE-afflicted chickens. Thus, it would have been detected if clinical or subclinical NE models were used to evaluate the efficacy of dietary encapsulated or powdered BP, as previously reported [8,9,12].

BP and their endolysins can reduce *C. perfringens* without disturbing the balance of the microbiota in the gastrointestinal tract [36]. Previous studies have shown that healthy chickens have a relatively low number of *C. perfringens* in the gastrointestinal tract [37,38]. Reference [39] reported that dietary powder-form BP decreased the cecal population of *C. perfringens* in non-challenged broiler chickens. In this study, *C. perfringens* challenge increased *C. perfringens* counts in cecal digesta on days 1 and 2 following *C. perfringens* challenge. Although dietary BP decreased *C. perfringens* in the cecal digesta, there were no differences in the reduction in *C. perfringens* counts between the encapsulated and powdered BP. The latter finding might be related to the current experimental design, as we freshly prepared the experimental diets and all birds exhibited superior growth. Thus, dietary BP would be equally effective in inhibiting *C. perfringens* without the advantage of encapsulation over the powder form.

The intestinal health of chickens can be determined by the balance between the anatomical components and their physiological activities [40]. The intact mucosa and intestinal villi and their microvilli are of utmost importance for adequate absorption of nutrients and the establishment of the intestinal microbiota, whereas the gut-associated mucosa provides an immune complex that functions as a gastric defense mechanism [40,41,42,43]. *C. perfringens* challenge has been reported to decrease the intestinal villus height and the ratio of villus height to crypt depth [35,44]. A higher ratio of villus height to crypt depth has been linked to a greater capacity for nutrient absorption [45,46] whereas deeper crypts indicate faster cellular turnover in response to inflammation induced by pathogens or toxins [35,47]. In line with earlier studies [44,48], we found that *C. perfringens* challenge increased crypt depth but decreased villus height to crypt depth ratio, indicating compromised gut health. Furthermore, dietary encapsulated or powdered BP lowered crypt depth but increased villus height to crypt depth ratio compared with the challenged POS group. These results suggest that dietary BP supplementation alleviates *C. perfringens*-altered gut morphology in broiler chickens.

SCFAs play a key role in the development of microflora in the ceca of growing broiler chickens [49]. *C. perfringens* challenge has been known to alter the cecal microbiota and intestinal SCFAs in broiler chickens. In particular, [50] found that *C. perfringens* challenge caused significant increases in *Clostridiaceae*, *Bacteroides*, and *Streptococcus*/*Lactococcus* counts in the crop, ileum, and cecal digesta. In addition, *C. perfringens* altered the concentration of cecal SCFAs in broiler chickens [1]. In this study, *C. perfringens* tended to lower total SCFAs in chickens, whereas dietary BP partially increased it. However, the observation that none of the treatments affected the relative percentages of SCFA suggests the negligible role of dietary BP in gut metabolites in the current study. Further studies are warranted to investigate the effects of BP intervention on changes in the gut microbiota in clinical or subclinical NE-afflicted chickens.

Chickens produce three classes of antibodies: IgY, IgM, and IgA [51] and IgA has two forms: serum IgA and secretory IgA (sIgA) [52]. Elevated concentrations of serum IgA have been shown to correlate well with higher sIgA in the intestine and may explain the mechanism of *C. perfringens* reduction in the intestinal lumen [53,54]. In addition, higher concentrations of serum IgG, IgA, and sIgA in gut digesta are known to lower the severity of pro-inflammatory responses [40,55]. IgA protects mucosal surfaces against toxins, viruses, and bacteria by neutralizing or preventing these pathogens from binding to the mucosal surface [10]. In this study, the concentrations of IgA in serum samples were lowest in the *C. perfringens*-challenged control group but highest in the encapsulated BP group. In line with our findings, [10] reported that *C. perfringens* challenge decreased serum IgA concentrations in broiler chickens compared to the non-challenged control group.

## 5. Conclusions

In this study, we confirmed the superior stability of in-feed encapsulated BPs compared to powder BPs in various segments of the gastrointestinal tract. In a *C. perfringens*-challenged broiler model, dietary BP supplementation, either in powder or encapsulated form, improved gut morphology (i.e., decreased crypt depth and increased villus height to crypt depth ratio), reduced *C. perfringens* counts in cecal digesta, and increased the concentration of IgA in serum samples compared with the *C. perfringens*-challenged control group. However, further studies are necessary to evaluate the effects of encapsulated BPs under harsh conditions (i.e., high humidity) and in a *C. perfringens*/*Eimeria* spp. dual-challenge NE model.

## Figures and Tables

**Table 1:** Ingredients in and chemical composition of the basal diet (%, as-fed basis).

Item	Content
Ingredients	
Corn	56.66
Soybean meal	29.00
Corn gluten meal	7.00
Animal fat	2.00
Iodized salt	0.30
Monocalcium phosphate	1.30
_DL_-methionine, 99%	0.35
_L_-lysine, 56%	0.50
_L_-threonine, 99%	0.10
Ground limestone	1.90
Sodium bicarbonate	0.24
Choline chloride, 50%	0.20
Vitamin premix ^1^	0.20
Mineral premix ^2^	0.25
Total	100.0
Calculated nutrient composition, %	
Nitrogen-corrected apparent metabolizable energy, kcal/kg	3039
Dry matter	87.9
Crude protein	22.2
Calcium	1.02
Total phosphorus	0.71
Available phosphorus	0.45
Chloride	0.21
Sodium	0.21
Lysine	1.33
Methionine	0.72
Methionine + Cysteine	1.08
Threonine	0.92
Arginine	1.29
Histidine	0.55

^1^ Vitamin mixture provided the following nutrients per kilogram of diet: vitamin A, 9000 IU; vitamin D3, 4000 IU; vitamin E, 58 mg; vitamin K3, 2.7 mg; vitamin B1, 2.3 mg; vitamin B2, 5.9 mg; vitamin B5, 17 mg; vitamin B6, 2.9 mg; vitamin B12, 0.015 mg; Niacin, 54 mg; Folic, 1.7 mg; biotin, 0.16 mg. ^2^ Mineral mixture provided the following nutrients per kilogram of diet: Mn, 85.7 mg; Cu, 100 mg; Zn, 64.3 mg; Fe, 57.1 mg; I, 0.57 mg; Co, 0.17 mg; Se, 0.2 mg.

**Table 2:** Effects of dietary encapsulated and powder bacteriophages (BPs) on the growth performance of broiler chickens before and after challenge with *Clostridium perfringens* ^1^.

Item ^3^	NEG	*C. perfringens* Challenge			SEM ^4^	*p*-Value
		POS	Powdered BP	Encapsulated BP		
BW, g/bird						
Day 0	41.01 ^2^	-	41.04	41.03	0.04	0.947
Day 7	179.5 ^2^	-	175.0	180.8	6.13	0.879
Day 14	519.4 ^2^	-	511.7	518.1	11.29	0.923
Day 21	1090.2 ^2^	-	1070.3	1095.1	16.85	0.730
Day 28	1822.9	1878.8	1801.4	1877.8	39.83	0.421
BWG, g/bird						
Day 0 to 7	138.4 ^2^	-	133.9	139.8	6.14	0.879
Day 7 to 14	339.9 ^2^	-	336.7	337.3	5.82	0.938
Day 14 to 21	570.8 ^2^	-	558.7	577.0	7.50	0.466
Day 21 to 28	744.4	776.9	731.0	782.8	21.34	0.271
FI, g/bird						
Day 0 to 7	129.1 ^2^	-	130.7	130.3	6.56	0.989
Day 7 to 14	372.4 ^2^	-	374.6	372.8	8.26	0.988
Day 14 to 21	692.6 ^2^	-	680.2	707.7	10.51	0.434
Day 21 to 28	995.0	1044.0	1017.6	1023.3	24.77	0.583
FCR, g:g						
Day 0 to 7	0.931 ^2^	-	0.976	0.929	0.018	0.313
Day 7 to 14	1.094 ^2^	-	1.112	1.104	0.008	0.437
Day 14 to 21	1.213 ^2^	-	1.218	1.226	0.009	0.707
Day 21 to 28	1.338	1.344	1.393	1.314	0.025	0.189

Abbreviations: BW, body weight; BWG, body weight gain; FI, feed intake; FCR, feed conversion ratio. ^1^ Values are least-squares means of 8 replicates unless otherwise stated. ^2^ Values are least-squares means of 16 replicates. ^3^ NEG = unchallenged group; POS = *C. perfringens* challenged control group; Powdered BP = challenged group fed diets supplemented with powdered BP; encapsulated BP = challenged group fed diets supplemented with encapsulated BP. ^4^ SEM, standard error of the means.

**Table 3:** Effects of dietary encapsulated and powder BPs on cecal *C. perfringens* counts (log_10_ cfu/g digesta) in broiler chicken challenged with *C. perfringens* ^1^.

Item ^2^	NEG	*C. perfringens* Challenge			SEM ^3^	*p*-Value
		POS	Powdered BP	Encapsulated BP		
Day 1 post *C. perfringens* challenge	4.75 ^b^	5.52 ^a^	5.05 ^b^	4.82 ^b^	0.15	0.008
Day 2 post *C. perfringens* challenge	4.22 ^b^	5.04 ^a^	4.40 ^b^	4.38 ^b^	0.20	0.047

^a,b^ Means without a common superscript letter differ (*p* < 0.05). ^1^ All means are average of 8 pens per treatment. ^2^ NEG = unchallenged group; POS = *C. perfringens* challenged control group; Powdered BP = challenged group fed diets supplemented with powdered BP; encapsulated BP = challenged group fed diets supplemented with encapsulated BP. ^3^ SEM, standard error of the means.

**Table 4:** Effects of dietary encapsulated and powder BPs on the ileal morphology of broiler chicken challenged with *C. perfringens* ^1^.

Item ^2^	NEG	*C. perfringens* Challenge			SEM ^3^	*p*-Value
		POS	Powdered BP	Encapsulated BP		
1 d post *C. perfringens* challenge						
Villus height (VH), µm	781.90	736.20	731.44	715.24	28.93	0.510
Crypt depth (CD), µm	128.83 ^b^	145.75 ^a^	120.67 ^b^	123.93 ^b^	5.09	0.004
VH: CD ratio, µm: µm	6.20 ^a^	5.17 ^b^	6.14 ^a^	5.87 ^a^	0.20	0.003

^a,b^ Means without a common superscript letter differ (*p* < 0.05). ^1^ All means are average of 8 pens per treatment. ^2^ NEG = unchallenged group; POS = *C. perfringens* challenged control group; Powdered BP = challenged group fed diets supplemented with powdered BP; encapsulated BP = challenged group fed diets supplemented with encapsulated BP. ^3^ SEM, standard error of the means.

**Table 5:** Effects of dietary encapsulated and powder BPs on the absolute or relative concentrations (mmol/kg digesta, % of total) of cecal short-chain fatty acids (SCFA) in broiler chicken challenged with *C. perfringens* ^1^.

Item ^2^	NEG	*C. perfringens* Challenge			SEM ^4^	*p*-Value
		POS	Powdered BP	Encapsulated BP		
mmol/kg						
Acetate	66.99	60.81	74.42	86.46	6.78	0.082
Propionate	5.86	4.82	6.21	6.42	0.69	0.402
Isobutyrate	0.89	0.99	0.97	0.98	0.13	0.957
Butyrate	17.68	14.62	22.75	23.37	3.19	0.223
Isovalerate	8.12	6.71	10.44	10.73	1.47	0.223
Valerate	1.29	1.19	1.41	1.72	0.21	0.352
Lactate	1.61	1.29	1.15	1.76	0.33	0.629
BCFA ^3^	10.30	8.90	12.82	13.43	1.56	0.193
SCFA ^3^	102.43	90.45	117.35	131.43	10.09	0.054
% of total SCFA						
Acetate	65.73	67.73	63.74	65.83	2.64	0.811
Propionate	5.92	5.30	5.33	4.99	0.57	0.773
Isobutyrate	0.93	1.20	0.85	0.77	0.18	0.393
Butyrate	16.85	15.78	19.12	17.64	1.94	0.729
Isovalerate	7.74	7.25	8.78	8.10	0.89	0.729
Valerate	1.31	1.31	1.19	1.33	0.16	0.941
Lactate	1.52	1.44	0.98	1.35	0.25	0.578
BCFA ^3^	9.98	9.76	10.82	10.20	0.86	0.879

^1^ All means are average of 8 pens per treatment. ^2^ NEG = unchallenged group; POS = *C. perfringens* challenged control group; Powdered BP = challenged group fed diets supplemented with powdered BP; encapsulated BP = challenged group fed diets supplemented with encapsulated BP. ^3^ SCFA, short-chain fatty acid (acetate + propionate + butyrate + isobutyrate + isovalerate + valerate + lactate); BCFA, branched-chain fatty acid (isobutyrate + valerate + isovalerate). ^4^ SEM, standard error of the means.

**Table 6:** Effects of dietary encapsulated and powder BPs on serum parameters in broiler chicken challenged with *C. perfringens* ^1^.

Item ^2^	NEG	*C. perfringens* Challenge			SEM ^4^	*p*-Value
		POS	Powdered BP	Encapsulated BP		
TAC ^3^, mM	0.367	0.480	0.464	0.478	0.038	0.187
NO, µM	22.38	22.80	17.02	21.17	1.89	0.230
IgA, mg/dL	21.22 ^ab^	18.06 ^b^	20.76 ^ab^	25.91 ^a^	1.76	0.046
CORT, pg/mL	120.92	136.81	106.35	102.81	8.21	0.154
TCHO, mg/dL	104.14	108.57	108.86	117.57	3.69	0.101
TG, mg/dL	43.00	44.75	51.50	55.75	7.73	0.633
GPT, U/L	3.00	3.00	2.75	3.38	0.26	0.432
GOT, U/L	211.43	203.57	193.86	210.0	11.61	0.703
HDL-C, mg/dL	91.00	88.00	89.29	97.25	3.32	0.253

^a,b^ Means without a common superscript letter differ (*p* < 0.05). ^1^ All means are average of 8 pens per treatment. ^2^ NEG = unchallenged group; POS = *C. perfringens* challenged control group; Powdered BP = challenged group fed diets supplemented with powdered BP; encapsulated BP = challenged group fed diets supplemented with encapsulated BP. ^3^ TAC, total antioxidant capacity; NO, nitric oxide; IgA, immunoglobulin A; CORT, corticosterone; TCHO, total cholesterol; TG, triglyceride; GPT, glutamic pyruvic transaminase; GOT, glutamic oxaloacetic transaminase; HDL-C, high-density lipoprotein-cholesterol. ^4^ SEM, standard error of the means.

## Data Availability

Not applicable.
